# Evaluation of the Protective Effects of Moringa oleifera, Zingiber officinale, and Allium sativum in a Rat Model of Chronic Kidney Disease (CKD)

**DOI:** 10.7759/cureus.84856

**Published:** 2025-05-26

**Authors:** Manjiri P Jalgaonkar, Rajeshwari K Bale, Alok D Singh, Gaurav M Doshi, Radhika K Raheja, Yogesh A Kulkarni, Manisha J Oza

**Affiliations:** 1 Quality Assurance, Shri Vile Parle Kelavani Mandal's (SVKM) Dr. Bhanuben Nanavati College of Pharmacy, Mumbai, IND; 2 Pharmacology, Shri Vile Parle Kelavani Mandal's (SVKM) Dr. Bhanuben Nanavati College of Pharmacy, Mumbai, IND; 3 Pharmacology, Shobhaben Pratapbhai Patel School of Pharmacy and Technology Management, Shri Vile Parle Kelavani Mandal's (SVKM) Narsee Monjee Institute of Management Studies (NMIMS) (Deemed to be University), Mumbai, IND; 4 Cardiovascular Research, Heart and Vascular Institute, Penn State College of Medicine, Penn State University, Hershey, USA

**Keywords:** adenine, allium sativum, chronic kidney disease, moringa oleifera, natural products, zingiber officinale

## Abstract

Background: Oxidative stress plays a key role in the development and progression of chronic kidney disease (CKD). In this study, it has been hypothesized that the extract of *Moringa oleifera* leaves, *Zingiber officinale* rhizomes, and *Allium sativum* cloves together might exert a protective effect against adenine-induced CKD in rats.

Methods: Rats were randomly divided into five groups: Group 1: normal control; Group 2: disease control; Group 3: CKD + polyherbal extract (250 mg/kg); Group 4: CKD + polyherbal extract (500 mg/kg); Group 5: CKD + polyherbal extract (1500 mg/kg). CKD was induced by feeding rats a 0.3% adenine-mixed diet for 28 days. Polyherbal extract was administered to rats, except for Groups 1 and 2, for 28 days.

Results: Experimental animals receiving treatment with polyherbal extract showed significant improvement in water intake (p< 0.001), urine volume (p< 0.01), urine creatinine (p < 0.05), plasma creatinine (p < 0.01), and blood urea nitrogen(p<0.05). Furthermore, the combined extract showed a potential effect in reducing oxidative stress and fibrosis, indicated by reduced hypertrophy in kidney tissue.

Conclusion: Polyherbal extract containing *Moringa oleifera* leaves, *Zingiber officinale* rhizomes, and *Allium sativum* clove extracts showed a reduction in fibrosis and oxidative stress, and thus, this polyherbal extract combination could be explored in-depth for its therapeutic potential in CKD.

## Introduction

Kidneys play a significant role in maintaining homeostasis by filtering, reabsorbing, secreting, synthesizing, and degrading metabolites through a variety of routes and help in removing toxins from the body; thus, disruption in the normal functioning of kidneys can have serious adverse effects on health [1]. Chronic kidney disease (CKD) is a condition characterized by abnormal kidney function and structure that persists for more than three months. It includes (1) a glomerular filtration rate < 60 mL/min/1.73 m² and (2) albuminuria, i.e., urine albumin 30 mg/ or a urine 24-hour albumin-to-creatinine ratio (ACR) ≥ 30 mg/g. The less common symptoms involve foamy urine, nocturia, hematuria, and a decrease in urine output [2]. CKD is a rapidly growing concern with few treatment options available, which inflicts a financial and emotional burden on the community [3]. Since 1990, CKD has been considered a non-communicable condition studied by the Global Burden of Disease. With the disease's growth rate accelerating, it has become a global concern [4]. The percentage of the population affected by CKD ranges from 8% to 16%, and it is a condition that patients and medical practitioners often fail to recognize. CKD can progress to end-stage renal disease (ESRD) and renal failure [5]. The pathogenesis involves oxidative stress and inflammation. Increased oxidative stress leads to the activation of pro-inflammatory cytokines [6].

*Moringa oleifera*, also called the ‘miracle tree,’ belongs to the family *Moringaceae*. Leaves of moringa are a rich source of vitamins [7] and flavonoids [8]. The major flavonoids include quercetin, myricetin, and kaempferol [9]. It has been reported that quercetin is also found in the form of quercetin-3-O-β-d-glucoside, also called isoquercetin; moringa is also rich in alkaloids, glucosinolates, isothiocyanates, tannins, and saponins [10]. *Zingiber officinale*, commonly called ginger, belonging to the family *Zingiberaceae*, has shown a variety of pharmacological activities, such as antimicrobial [11], antioxidant [12], and anti-inflammatory [13] activities, and cardiovascular diseases [14] and neurodegenerative diseases [15]. The major active compound is ginger gingerol [16]. *Allium sativum*, commonly called garlic, belongs to the family, is widely known for its culinary purposes [17]. It has also shown protective effects against certain conditions in studies. A growing phenomenon that has been receiving attention lately is the process of converting fresh garlic into black garlic. Black garlic is a processed form of fresh garlic obtained by subjecting fresh garlic to high temperatures in the presence of humidity for a few weeks or even months [18]. The process of fermentation changes the color, taste, and smell of garlic [19]. Black garlic has a dark color, lacks the characteristic pungent smell of garlic, is sweet, and is chewy or jelly-like in consistency. These changes are attributed to the alterations in the phytoconstituents present in garlic. Allicin, responsible for the strong flavor of garlic, is converted to S-allyl cysteine (SAC), which is also considered a major sulfur compound in black garlic. SAC, S-allyl-mercapto cysteine, 5-hydroxymethylfurfural (5-HMF), organosulfur compounds, and polyphenols are some of the compounds present in high quantities in black garlic as compared to fresh garlic. During the aging process, because of the Maillard reaction, 5-HMF, and 5-(hydroxymethyl-2)-furoic acid (5-HMFA) are produced. 5-HMF is reported to be associated with the black color in black garlic. Studies have shown that phenolic and flavonoid-like compounds increase during the aging process [20]. *Moringa oleifera* leaves, *Zingiber officinale* rhizomes, and *Allium sativum* (black garlic) extracts are studied for their nephroprotective potential individually. These herbs are also reported as antioxidant and anti-inflammatory agents in various other studies. Even the polyherbal extract of these three herbs has been reported to reduce oxidative stress and inflammation and improve immunity [21, 22]. However, there are no reports available to show the combined effect of the extract prepared from *Moringa oleifera* leaves and *Zingiber officinale* rhizomes with black garlic extract in CKD. Thus, the current study was designed to understand, explore, and evaluate the effects of the extract of *Moringa oleifera* leaves, *Zingiber officinale *rhizomes, and *Allium sativum* extracts on the adenine-induced CKD rat model.

## Materials and methods

Chemicals

Adenine was procured from Tokyo Chemical Industry (India) Pvt. Ltd., Chennai, India. Diagnostic kits were procured from Transasia Bio-medicals Ltd., India, Bangalore, India.

Collection and authentication of plant materials

*Moringa oleifera* leaves were collected from Keshav Srushti, Thane, India. *Zingiber officinale* rhizomes were procured from Satara, India. *Allium sativum* was obtained through a vendor from Kashmir, India. *Moringa oleifera* (MIT0173), *Zingiber officinale *(MIT0174), and *Allium sativum* (MIT0175) were authenticated at Mithibai College of Arts, Science and Commerce, Mumbai, India.

Preparation of extracts

*Moringa oleifera* leaves were thoroughly washed and dried in a hot air oven. The dried leaves were ground into powder in a multimill. Extraction was carried out by the cold maceration technique by using 70% ethanol for 72 hours. The process was repeated three times to obtain exhaustive extraction. The dried ginger rhizomes were ground into powder in a multi-mill. The powder was extracted by using 95% ethanol by the cold maceration technique for 72 hours. The process was repeated thrice to obtain exhaustive extraction. Fresh *Allium sativum *was peeled and subjected to heat treatment for one month by keeping the garlic in a rice cooker. Garlic was checked at regular intervals for the completion of the blackening process. Black garlic was ground in a mixer to obtain a paste. The paste was macerated with 95% ethanol for 72 hours. The process was repeated thrice to obtain exhaustive extraction. The resulting extracts were then pooled and filtered, and the filtrate was concentrated at reduced pressure using a Rota evaporator at 50ºC-60ºC, and the resulting extract was stored at 4ºC until further use.

Total phenolic and flavonoid contents

The total phenolic content of the *Moringa oleifera* leaves, *Zingiber officinale *rhizomes, and *Allium sativum* clove extracts was determined by using the Folin-Ciocalteu colorimetric method. In brief, the Folin-Ciocalteu reagent was diluted 10-fold with distilled water. 1 ml of extract or standard was mixed with 5 ml of Folin-Ciocalteu reagent and allowed to stand for 10 minutes at room temperature. Then, 4 ml of 7.5% sodium carbonate (weight per volume (w/v)) solution was added, and this mixture was allowed to stand in the dark for 45 minutes. The absorbance was measured at 765 nm using a UV-visible spectrophotometer. The calibration curve was prepared by using gallic acid as a standard in water and methanol [23]. The purpose of the study was to use the aluminum chloride colorimetric method to measure the number of flavonoids present in extracts from* Zingiber officinale* rhizomes, *Allium sativum*, and *Moringa oleifera* leaves. This was accomplished by mixing 1.5 ml of methanol, 0.3 ml of a 5% w/v sodium nitrite solution, and 1 ml of either the test or reference sample. 0.5 cc of 2% aluminum chloride was added after the liquid had stood for five minutes, and the sample was well combined. After six minutes, 0.5 ml of 1 M potassium acetate was added to neutralize the mixture. A UV-visible spectrophotometer was used to detect the absorbance at 510 nm after the samples had been at room temperature for 10 minutes. Quercetin was used to generate a calibration curve as the standard in methanol [24].

Standardization of the extracts by using high-performance liquid chromatography (HPLC)

Reverse-phase chromatography technique and a water HPLC system were used for analysis. The dimensions of the analytical column used were 5 μm and 250×4.6 mm. The column temperature was set at 25ºC, and the injection volume was 20 μl. Isocratic elution was used, and the mobile phase consisted of acetonitrile and water in a ratio of 70:30. The flow rate was set at 1 ml/min, and the run time was 10 minutes. The wavelength for analysis was set at 255 nm. A standard quercetin solution was prepared and injected, followed by the preparation of *Moringa oleifera* leaves, *Zingiber officinale* rhizomes, and *Allium sativum* extract solutions for HPLC. Various concentrations of standard quercetin solutions were prepared (50, 100, 150, 200, 250, 300, 350, and 400 ppm) and injected to obtain a standard curve of quercetin. A calibration curve of quercetin was prepared by plotting the concentration against the area under the curve. This quercetin standard curve was used to quantify the quercetin present in *Moringa oleifera* leaves, *Zingiber officinale* rhizomes, and *Allium sativum* extracts.

Experimental animals

The study was conducted at the Department of Quality Assurance and the Department of Pharmacology, Shri Vile Parle Kelavani Mandal's (SVKM) Dr. Bhanuben Nanavati College of Pharmacy, Mumbai, India. Approval (CPCSEA/IAEC/P-08/2022) was taken from the Institutional Animal Ethics Committee (IAEC) before initiating the experiment. Sprague Dawley rats were procured from the National Institute of Biosciences, Pune, Maharashtra, India. The animals were acclimatized to laboratory conditions for one week before the initiation of the experiment.

Induction of CKD in experimental animals

Each of the five groups, namely, normal control, disease control, disease + polyherbal extract at 250 mg/kg, disease + polyherbal extract at 500 mg/kg, and disease + polyherbal extract at 1500 mg/kg, was randomly allocated to the animals, which weighed between 150 and 180 grams. In order to cause CKD, the other four groups were fed 0.3% adenine in their feed for 28 days [25], while the normal control group was given a regular diet. For an additional 28 days after the adenine treatment, the animals in Groups 3, 4, and 5 received oral gavages of polyherbal extract at doses of 250 mg/kg, 500 mg/kg, and 1500 mg/kg, respectively. The dose selection was done based on pharmacological and toxicological reports available in the literature for individual extracts as well as for the polyherbal extracts of these herbs. Several evaluation factors were evaluated following this treatment period to ascertain the impact of the polyherbal extract.

Evaluation parameters

Body Weight and Water Intake

Once a week, the animals' body weight and water intake were recorded.

Urine and Biochemical Parameters

The individual animal was kept in a metabolic cage (BIK Industries, Mumbai, India) for the collection of urine. The amounts of albumin, creatinine, and total protein were among the indicators that were examined in the urine. Furthermore, plasma samples were used to estimate biochemical parameters such as blood urea nitrogen (BUN) and creatinine levels. All of these parameters were assessed using diagnostic kits with the help of a biochemical analyzer (Erba Chem-7, Erba Diagnostics Mannheim GmbH, Mannheim, Germany).

Kidney Hypertrophy

To establish a systematic assessment of kidney size relative to total body mass, the study entailed euthanizing the animals and extracting their kidneys, which were subsequently weighed and divided by their body weight to calculate their relative kidney weight.

Estimation of Oxidative Stress Markers in Renal Tissue

The oxidative stress in the kidney tissue was assessed by measuring malondialdehyde (MDA) and reduced glutathione (GSH) levels.

Histopathological Analysis of Kidney Tissue

Periodic acid Schiff (PAS) stain was used to assess the histopathological changes in the kidney tissue. The renal tissue was embedded in paraffin wax, and thin sections were stained and observed under a microscope.

Statistical Analysis

GraphPad Prism (GraphPad Software, La Jolla, CA) was used to analyze the data. All data are expressed as mean ± SEM. The data were assessed by using one-way ANOVA followed by Dunnett’s multiple comparison. p < 0.05 was considered to be significant.

## Results

Total phenolic and flavonoid contents

Phenolic acids contribute highly to the antioxidant activity, and hence, the total phenolic content in plants is determined. The Folin-Ciocalteu method was used to estimate the phenolic content in the extracts. The results are presented in Table 1 as mg/g gallic acid equivalents (GAE) of the extract. The extractive value of moringa leaf extract, ginger extract, and black garlic was 1.09%, 1.35%, and 1.23%, respectively. Ginger extract exhibited the highest total phenolic content, whereas fresh garlic extract exhibited the lowest total phenolic content. A considerable increase in the phenolic content was observed after the blackening process in the black garlic extract. The aluminum chloride colorimetric method was used to estimate the total flavonoid content in the extracts; the results are presented in Table 1. Black garlic extract showed the highest phenolic content, whereas *Moringa oleifera* extract showed the lowest.

**Table 1:** Total phenolic and flavonoid contents QE: quercetin equivalent; GAE: gallic acid equivalents

Sr. No.	Extracts	Total phenolic content (mg/g GAE)	Total flavonoid content (mg/g QE)
1	*Moringa oleifera *extract	55.35 ± 5.09	25.33 ± 13.61
2	*Zingiber officinale* extract	100.23 ± 7.10	41.15 ± 6.07
3	Fresh garlic (*Allium sativum*) extract	40.17 ± 9.11	217.48 ± 7.23
4	Black garlic (*Allium sativum*) extract	72.65 ± 2.40	264.72 ± 8.99

Standardization of the extracts by using HPLC

**Figure 1:**
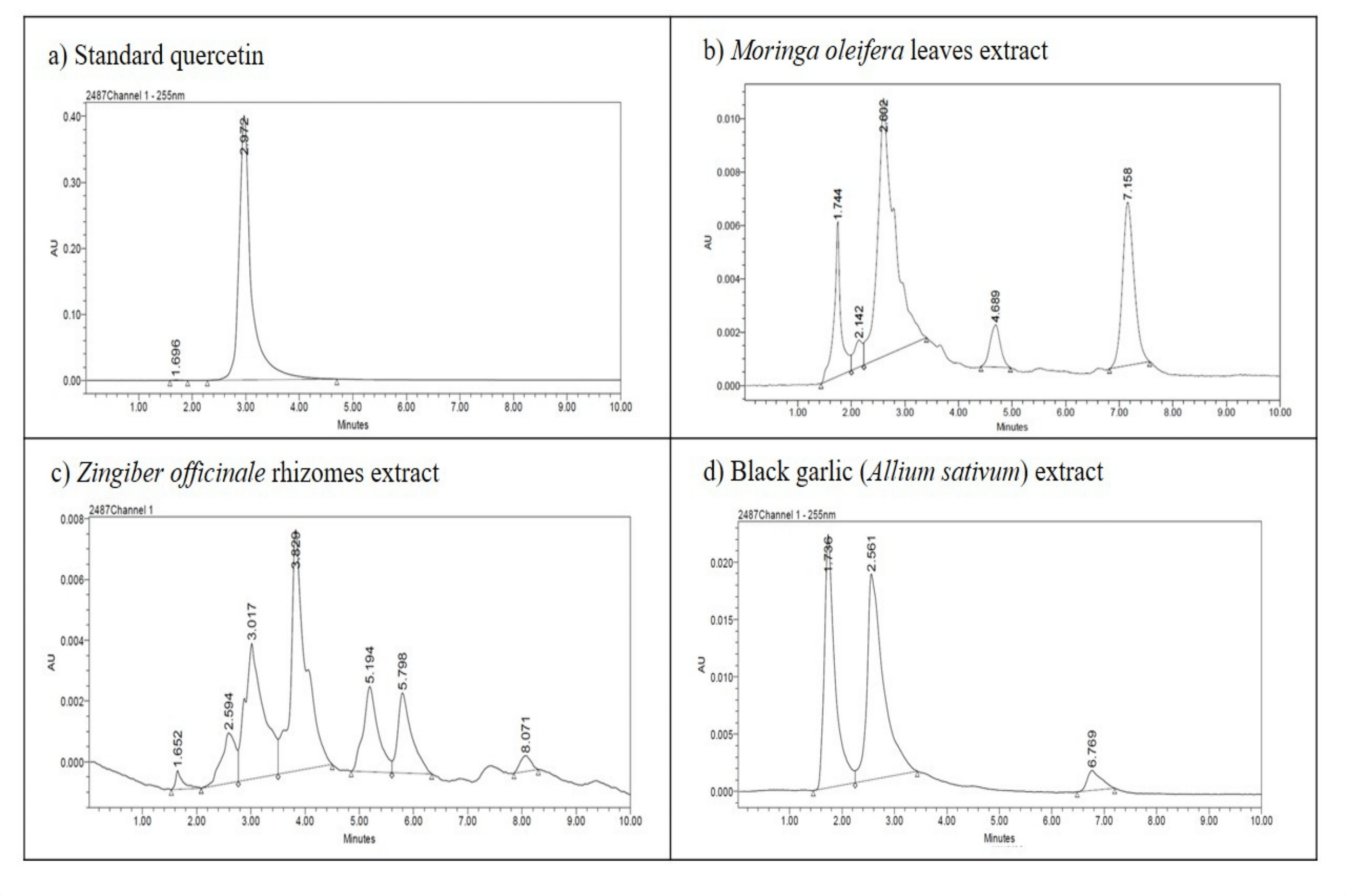
HPLC chromatogram of a) standard quercetin, b) Moringa oleifera leaf extract, c) Zingiber officinale rhizome extract, and d) black garlic (Allium sativum) extract. HPLC: high-performance liquid chromatography

**Figure 2:**
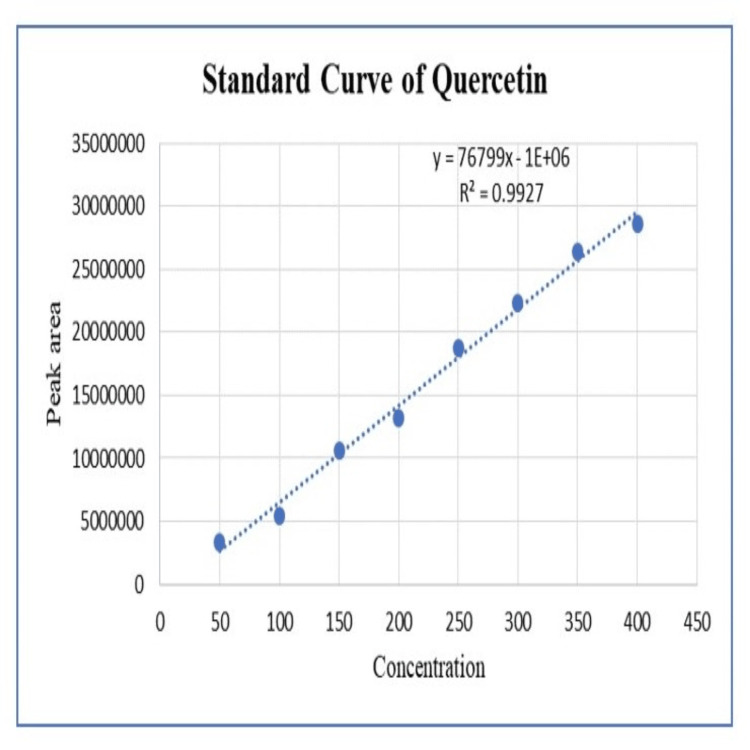
Standard curve of quercetin (HPLC) HPLC: high-performance liquid chromatography

The ethanolic extracts of *Moringa oleifera* leaves, *Zingiber officinale* rhizomes, and black *Allium sativum* extracts showed peaks at a similar retention time (RT) as that of standard quercetin (RT=2.9 minutes), which confirmed the presence of quercetin in the extracts. *Moringa oleifera* leaf extract showed a prominent peak at 2.6 minutes, *Zingiber officinale* rhizome extract showed a peak at 3.0 minutes, and black *Allium sativum* extract showed a peak at 2.5 minutes. The HPLC chromatogram is shown in Figure 1. A standard curve of quercetin was prepared by plotting the concentration of standard quercetin against the peak area shown in Figure 2. The regression equation was y = 76799x - 1E+06 and R² = 0.9927, which was used to calculate the amount of quercetin present in the extracts; it has been summarized in Table 2.

**Table 2:** Concentration of quercetin in extracts

Sr. No.	Extract	Concentration of quercetin (mg/g)
1	*Moringa oleifera* extract	19.37 ± 1.28
2	*Zingiber officinale* extract	61.27 ± 5.84
3	*Allium sativum *(Black garlic) extract	71.12 ± 5.51

Effect of the polyherbal extract on body weight and water intake

**Figure 3:**
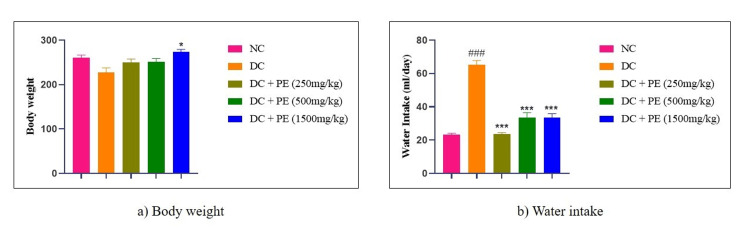
Effect of the PE on a) body weight and b) water intake The data were analyzed using one-way ANOVA followed by Dunnett’s multiple comparison test, and all values are expressed as mean ± SEM (n=6). ###p < 0.001, when compared with normal control. p<0.05, **p<0.001, when compared with disease control. NC: normal control; DC: disease control; PE: polyherbal extract; SEM: standard error of the mean

Every week, the animals' body weight was measured. Body weight decreased after adenine treatment, whereas body weight increased after the polyherbal formulation was administered at doses of 250 mg/kg and 500 mg/kg in comparison to the disease control group. Notably, as shown in Figure 3, animals that received the maximum dosage of 1500 mg/kg showed a substantial increase in body weight (*p<0.05). Furthermore, compared to the normal control group, adenine administration through diet was linked to a significant increase in water consumption (###p<0.001). On the other hand, water intake was considerably decreased by treatment with the polyherbal extract at doses of 250 mg/kg (***p<0.001), 500 mg/kg (***p<0.001), and 1500 mg/kg (***p<0.001).

Effect of the polyherbal extract on urine parameters

**Figure 4:**
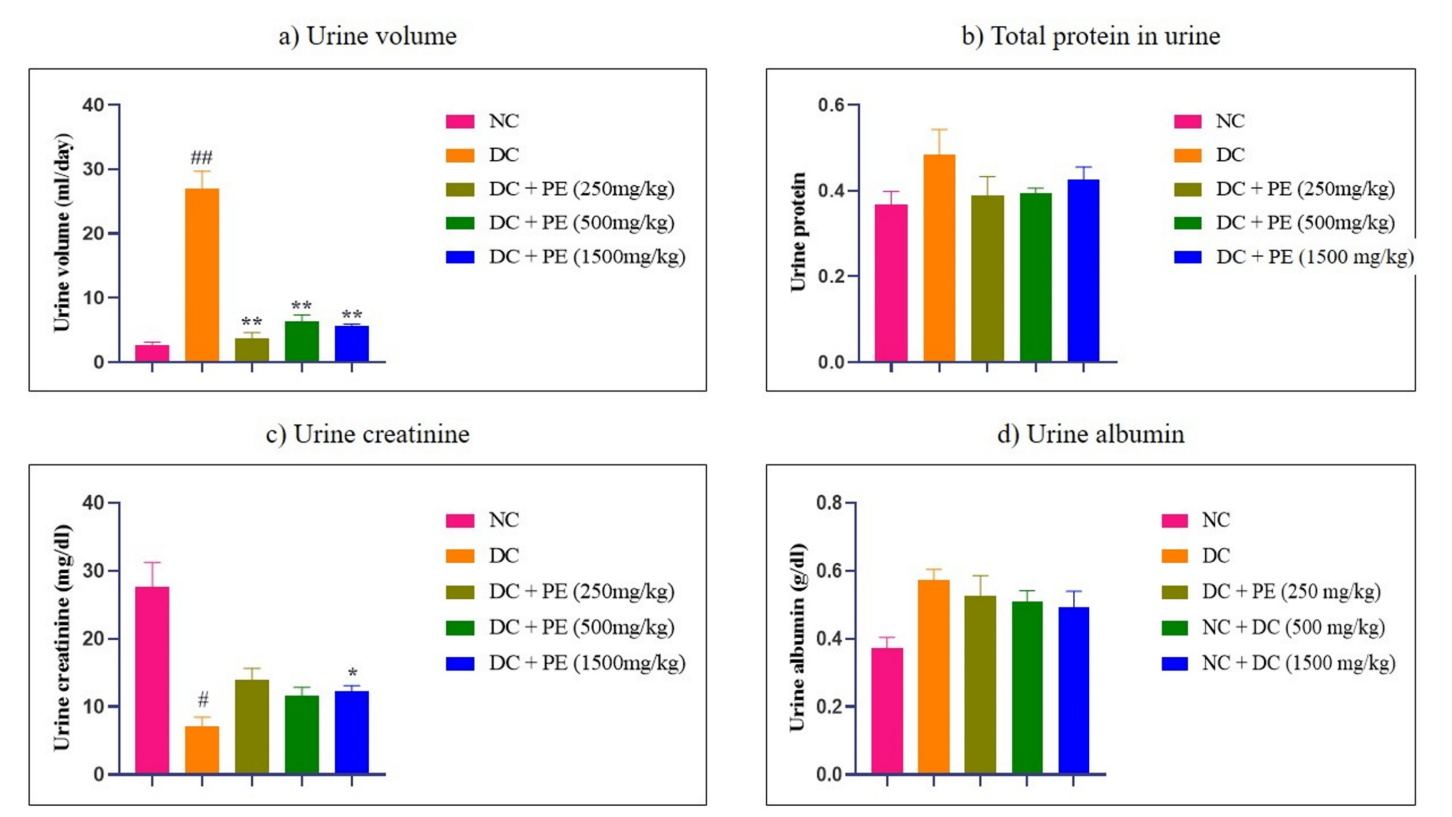
Effect of the PE on urine parameters a) Urine volume; b) Total protein in urine; c) Urine creatinine; d) Urine albumin The data were analyzed using one-way ANOVA followed by Dunnett’s multiple comparison test, and all values are expressed as mean ± SEM (n=6). #p<0.05, ##p<0.01, when compared with normal control. p<0.05, *p<0.01 when compared with disease control. NC: normal control; DC: disease control; PE: polyherbal extract; SEM: standard error of the mean

Animals given adenine showed a significant increase in urine production when compared to the normal control group. However, as shown in Figure 4a, the administration of the polyherbal extract at doses of 250 mg/kg (**p<0.01), 500 mg/kg (**p<0.01), and 1500 mg/kg (**p<0.01) resulted in a considerable decrease in urine volume. Urine protein levels were likewise higher in the adenine group than in the normal group, but the polyherbal formulation successfully reduced urine protein at all treatment doses (Figure 4b). Additionally, after taking adenine, creatinine excretion was considerably reduced. On the other hand, the highest dose of the polyherbal extract, 1500 mg/kg, caused a substantial increase in creatinine excretion (*p<0.05), and the 250 mg/kg and 500 mg/kg doses also caused the treated animals' urine creatinine levels to rise (Figure 4c). Furthermore, animals treated with adenine had higher urine albumin levels, while animals administered the polyherbal extract at doses of 250, 500, and 1500 mg/kg had lower urine albumin levels (Figure 4d).

Effect of the polyherbal extract on plasma creatinine and BUN

**Figure 5:**
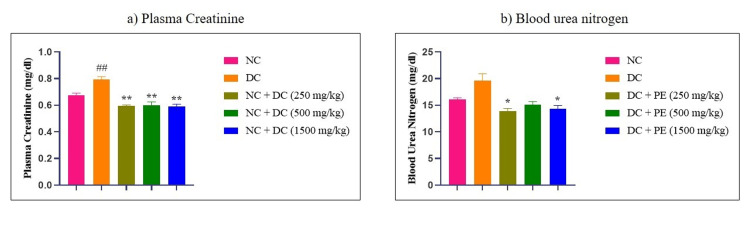
Effect of the PE on a) plasma creatinine and b) blood urea nitrogen The data were analyzed using one-way ANOVA followed by Dunnett’s multiple comparison test and all values are expressed as Mean ± SEM (n=6). ##p<0.01, when compared with normal control. *p<0.05, **p<0.01 when compared with disease control. NC: normal control; DC: disease control; PE: polyherbal extract; SEM: standard error of the mean

When compared to the normal group, the rats in the illness group had higher plasma creatinine levels after receiving adenine. In contrast, plasma creatinine levels in the treated animals were considerably lowered by administration with the polyherbal extract at all doses: 250 mg/kg (**p<0.01), 500 mg/kg (**p<0.01), and 1500 mg/kg (**p<0.01). Animals treated with adenine showed elevated BUN levels, which are a sign of kidney injury. However, as Figure 5 illustrates, after 28 days of therapy with the polyherbal extract, there was a significant drop in BUN levels, especially in the groups that received dosages of 250 mg/kg and 1500 mg/kg (*p<0.05).

Effect of the polyherbal extract on kidney hypertrophy

**Figure 6:**
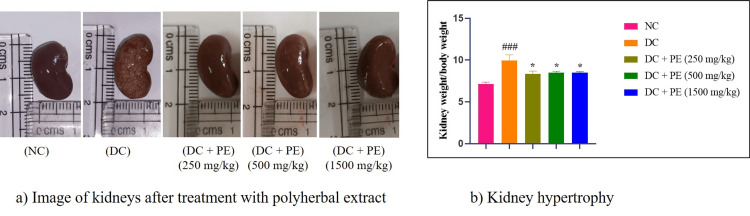
Effect of the PE on kidney hypertrophy a) Image of kidneys after treatment with the PE; b) Kidney hypertrophy. The data were analyzed using one-way ANOVA followed by Dunnett’s multiple comparison test, and all values are expressed as mean ± SEM (n=6). ###p<0.001 when compared with normal control. *p<0.05 when compared with disease control. NC: normal control; DC: disease control; PE: polyherbal extract; SEM: standard error of the mean

One measure of renal hypertrophy is relative kidney weight. In the study, the relative kidney weight of the adenine-treated rats was considerably higher than that of the normal group (###p<0.001). Relative kidney weight, however, significantly (*p<0.05) decreased after treatment with the polyherbal extract at doses of 250, 500, and 1500 mg/kg, when compared with disease control animals. Furthermore, adenine-treated animals' kidneys showed signs of damage and paleness, whereas the normal group's kidneys had a reddish-brown color (Figure 6).

Effect of the polyherbal extract on oxidative stress parameters

**Figure 7:**
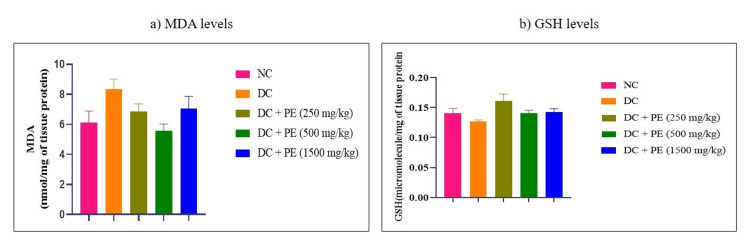
Effect of the PE on a) MDA levels and b) GSH levels The data were analyzed using one-way ANOVA followed by Dunnett’s multiple comparison test, and all values are expressed as Mean ± SEM (n=6). NC: normal control; DC: disease control; PE: polyherbal extract; SEM: standard error of the mean; MDA: malondialdehyde; GSH: glutathione

Compared to the normal group, rats treated with adenine had higher levels of MDA. However, MDA levels decreased in all treatment groups when the polyherbal formulation was administered. Furthermore, it was observed that rats treated with adenine had decreased levels of GSH, whereas all treatment groups showed an effective rise in GSH levels due to the polyherbal formulation (Figure 7).

Histopathology study of the kidney

**Figure 8:**
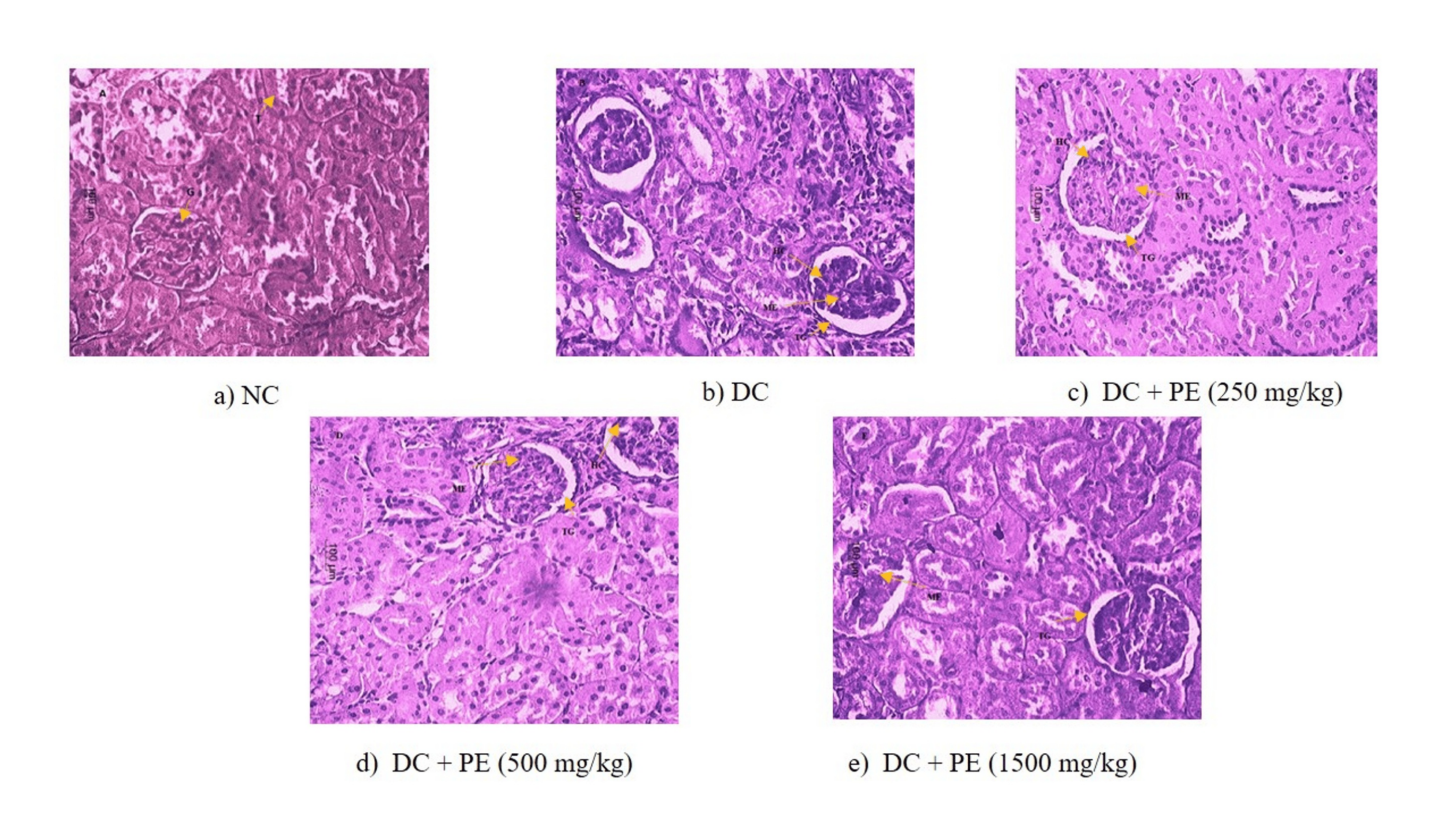
Effect of the PE on the histopathology of kidney tissues a) NC; b) DC; c) DC + PE (250 mg/kg); d) DC + PE (500 mg/kg); e) DC + PE (1500 mg/kg); The arrow indicates the glomerular basement membrane area. All sections were stained with PAS stain. NC: normal control; DC: disease control; PE: polyherbal extract; PAS: periodic acid-Schiff

The kidney tissues stained with PAS showed minimal to mild hypercellularity of mesangial cells in multiple areas, moderate to mild expansion of mesangial matrix in multiple areas, and mild to moderate thickening of glomerular basement membrane in some areas in the disease-control animals as compared to normal animals. However, treatment with the polyherbal extract resulted in a reduction of renal damage. The animals treated with the polyherbal extract (1500 mg/kg) showed a significant decrease in mesangial expansion, leukocyte infiltration, and glomerular basement thickening (Figure 8 and Table 3).

**Table 3:** Histopathological examination of the kidneys (PAS staining) Lesion severity was recorded as 0 = not present, 1 = minimal (<1%), 2 = mild (1–25%), 3 = moderate (26–50%), 4 = moderately severe (51–75%), 5 = severe (76–100%), and distribution was recorded as focal, multifocal, and diffuse. NC: normal control; DC: disease control; PE: polyherbal extract; PAS: periodic acid-Schiff

Lesions	NC	DC	DC + PE (250 mg/kg)	DC + PE (500 mg/kg)	DC + PE (1500 mg/kg)
Mesangial matrix expansion	0	4	4	3	3
Focal minimal	0	1	0	1	1
Multifocal minimal	0	0	1	1	1
Multifocal mild	0	1	1	0	1
Multifocal moderate	0	1	2	0	0
Diffuse mild	0	1	0	1	0
Diffuse moderate	0	0	0	0	0
Mesangial cell hypercellularity	0	4	3	3	0
Focal mild	0	1	0	0	0
Multifocal minimal	0	1	2	1	0
Multifocal mild	0	2	1	2	0
Thickening of glomerular basement membrane	0	4	3	3	1
Focal minimal	0	0	3	0	0
Focal mild	0	1	0	2	0
Multifocal minimal	0	2	0	1	1
Multifocal mild	0	0	0	0	0
Multifocal moderate	0	1	0	0	0

## Discussion

Despite the advancements in intervention techniques, the incidence, prevalence, and cost of treating CKD are increasing, which suggests that research into new pharmacological agents for CKD should be encouraged. *Moringa oleifera* leaves, *Zingiber officinale* rhizomes, and *Allium sativum* have been individually shown to have positive effects, including antihyperglycemic, antioxidant, antihyperlipidemic, and anti-inflammatory effects, many of which have been considered to be the underlying cause in the development of CKD. Similarly, the beneficial properties of garlic have been reported to be enhanced after it is processed by heating and fermentation to convert it into black garlic [20]. The medicinal properties shown by these herbs are due to the presence of a variety of active compounds. Ginger is most commonly known for the presence of gingerols and shogaols, whereas black garlic is famous for its sulfur-containing compounds. Moringa, on the other hand, has been called a miracle tree and consists of phytoconstituents ranging from alkaloids and tannins to vitamins [4]. Quercetin is one of the major phytoconstituents. Previous studies have exhibited the renoprotective effects of *Moringa oleifera*, *Zingiber officinale*, and *Allium sativum* [4,26,27]. Thus, it has been hypothesized in the present study that a polyherbal extract containing these three herbs might show a beneficial effect in reducing the complications of CKD. Different models have been used to induce CKD in animals, some of which include the 5/6 nephrectomy model, unilateral nephrectomy, streptozotocin (STZ)-induced CKD, one-kidney one-clip, two-kidney two-clip model, and adenine-induced CKD. The adenine-induced CKD model was selected as it mimics the structural and functional changes caused in humans suffering from CKD, some of which include serum creatinine level, increased serum urea nitrate, albuminuria, and increased oxidative stress [28, 25].

Kidney damage is caused by CKD, which is characterized by increased urine volume, increased levels of plasma creatinine, BUN, urine albumin, kidney hypertrophy, and decreased creatinine clearance. Kidney damage has also been reported to affect the water consumption of an individual. It increases the water intake, possibly because it interacts with the metabolic process by interfering with CYP450, which is a liver enzyme. Thus, biochemical and urine parameters such as urine output, urine albumin, urine creatinine, plasma albumin, BUN, etc., were estimated to understand the effect of the extract in CKD. Treatment with polyherbal extract resulted in improvement in the urine parameters as well as biochemical parameters. In the present study, adenine-treated animals showed increased levels of urine albumin, plasma creatinine, BUN, and urine total protein and decreased levels of urine creatinine. These disturbed levels were normalized by treatment with the polyherbal extract in the treatment group animals.

Another significant cause in the development and progression of CKD is oxidative stress. An increase in oxidative stress results in the generation of reactive oxygen species (ROS) and reactive nitrogen species (RNS), which in turn lead to cellular damage, inflammation in the kidney, oxidation of lipids, proteins, and DNA, and end in kidney injury [29, 25]. Animals receiving polyherbal extract also showed a decrease in oxidative stress, which can be confirmed by the increased GSH levels and decreased MDA levels in the treatment group animals as compared to the animals in the disease group.

## Conclusions

The present study is an attempt to understand the beneficial effects of *Moringa oleifera* leaves, *Zingiber officinale* rhizomes, and black *Allium sativum* in reducing the complications of CKD. Although the current findings are positive, it is crucial to explore further with respect to the pharmacokinetic profile of the combined extract and the mechanistic pathways involved and/or any synergistic effects that might be shown by the phytoconstituents present in *Moringa oleifera* leaves, *Zingiber officinale* rhizomes, and black *Allium sativum*.
